# In Vivo Monitoring of the Growth of Fertilized Eggs of Medaka Fish (*Oryzias latipes*) by Near-Infrared Spectroscopy and Near-Infrared Imaging—A Marked Change in the Relative Content of Weakly Hydrogen-Bonded Water in Egg Yolk Just before Hatching

**DOI:** 10.3390/molecules21081003

**Published:** 2016-08-01

**Authors:** Mika Ishigaki, Yui Yasui, Paralee Puangchit, Shoya Kawasaki, Yukihiro Ozaki

**Affiliations:** School of Science and Technology, Kwansei Gakuin University, Gakuen, Sanda, Hyogo 669-1337, Japan; yasuiyui@kwansei.ac.jp (Y.Y.); ambebeam@gmail.com (P.P.); shoya19921013@gmail.com (S.K.)

**Keywords:** NIR spectroscopy and imaging, in vivo monitoring, fertilized egg

## Abstract

The present study develops further our previous study of in vivo monitoring at the molecular level of the embryonic development in Japanese medaka fish (*Oryzias latipes*) using near-infrared (NIR) spectroscopy and NIR imaging. NIR spectra were measured nondestructively for three major parts of fertilized medaka eggs (the embryonic body, oil droplets, and egg yolk) from the first day after fertilization to the day just before hatching (JBH). Changes in the contents of chemical components such as proteins, water, and lipids were monitored in situ during embryonic development. A marked change in the relative content of weakly hydrogen-bonded water was observed in the egg yolk JBH. Principal component analysis (PCA) was carried out using the NIR spectra data of the egg yolk and embryo on the fifth day after fertilization. The PCA clearly separates the egg yolk data from the embryo body parts. Principal component PC1 and PC2 loading plots suggest that the hydrogen bonding structure of water in the egg yolk is considerably different to those of the other parts and the fraction of weakly hydrogen-bonded water in the egg yolk is smaller than that in the embryonic body. NIR images developed from the intensities of peaks of second derivative spectra owing to water and proteins show their different distribution patterns. Images of the ratio of strongly and weakly hydrogen-bonded water confirmed that oil droplets and embryonic body parts have higher and lower ratios, respectively, of strongly hydrogen-bonded water than do the other parts. The images developed from the intensity of the peaks at 4864 and 4616 cm^−1^ related to the proteins indicated that the egg yolk contains a higher concentration of protein than do the other parts. The peaks at 5756 and 4530 cm^−1^ caused by the protein secondary structures of α-helix and β-sheet showed the configuration of the egg cell membrane. The present study might lead to new understanding at the molecular level regarding the growth of fertilized eggs and provides a new tool to visualize egg development in a nondestructive manner.

## 1. Introduction

Studies of embryonic development are important in terms of understanding basic biology and potential applications. The majority of previous biological studies of embryonic development have been related to biochemical analysis of embryos. The variations of biochemical components such as DNA, proteins, and lipids have been investigated using various analytical tools. For example, the characteristic behavior of lipid dynamics and utilization of lipid components have been examined with staining, RNA sequencing, and gas chromatography [1,2,3,4,5]. Furthermore, quantitative analysis and the variation of proteins within embryos have been investigated using electrophoresis or mRNA assays [6,7]. Utilizing these different methods enables the detection of changes in the internal components of embryos, which are related to life stage activities and compositional differences caused by environmental factors. However, until quite recently it has not been possible to find a useful nondestructive analytical tool for monitoring embryonic development at the molecular level.

In terms of practical applications, embryonic quality has been investigated extensively in marine culture systems, the livestock industry, and fertilization treatments in humans. Embryonic quality has been shown to have a close relationship with the survival potential of an embryo. In human fertility treatments, it is well known that embryonic quality assessed by a grading method based on cleavage rates and the morphological features of the ovum are closely related to pregnancy success rates after in vitro fertilization [8,9,10,11,12]. In fish eggs, it has also been reported that the embryos with symmetric blastomeres, equal cell size, and complete cell margins have high hatching rates [13,14]. Morphological features are one of the predictive factors for embryonic survival potential; however, their analysis method appears to be empirical and phenomenalistic, and it does not provide any information about the exact embryonic quality.

From the perspectives of both basic biology and practical applications, it is essential to establish powerful nondestructive evaluation techniques that allow us to monitor embryonic development and quality in situ at the molecular level. In our previous study, we demonstrated the potential of near-infrared (NIR) spectroscopy and imaging in monitoring embryonic development and its quality in situ [15].

NIR imaging has received a great deal of attention because it demonstrates the distribution of components including in thick materials [16,17,18,19,20,21,22]. NIR imaging collects tens of thousands of spatially distinct NIR spectra simultaneously. These data provide qualitative and quantitative insight into the functionality of heterogeneous samples including pharmaceutical tablets, polymer laminates, and agricultural and biological materials. Because NIR imaging data are collected in a spatially resolved manner, it is possible to analyze them in different positions. NIR imaging is an in situ and nondestructive analysis method, which allows noncontact analysis and analysis using light fiber. Moreover, NIR imaging can be used to probe an aqueous dispersion more easily than via infrared imaging [23,24,25,26]. NIR light can penetrate deeper into a sample than infrared light can and, thus, NIR imaging can be utilized to easily probe thick samples or bulk materials with little or no sample preparation required. Therefore, NIR imaging is very suitable for biological materials, even for those containing large amounts of water.

In our previous study, we investigated the growing development of a fertilized medaka fish (Japanese killifish, *Oryzias latipes*) egg noninvasively using NIR spectroscopy and NIR imaging [15]. We were able to monitor separately the changes in the contents of egg proteins and lipids in the embryo, egg yolk, and oil droplets. Moreover, variations in their distribution were visualized using NIR imaging. The changes in the relative intensity of the NIR imaging bands because of proteins and lipids, as well as results from the principal component analysis (PCA), revealed a relative concentration change in proteins during the process of growth in the fertilized eggs. The present study aims to investigate in greater detail the changes in protein contents in the embryo, egg yolk, and oil droplets in medaka eggs, with new findings being reported. For example, we observed in the present study that the content of weakly hydrogen-bonded water varied markedly in the egg yolk just before hatching (JBH). Moreover, the new NIR imaging result developed by the intensity of the bands at 4864 and 4616 cm^−1^ caused by a combination of the NH stretching mode and amide II revealed that protein concentration in the egg yolk is higher than in the other parts. Egg membrane configurations can also be visualized using the bands caused by α-helix and β-sheet structures at 5756 and 4530 cm^−1^. The present study aimed to provide new insights into the growth of fish eggs at the molecular level.

## 2. Material and Methods

### 2.1. Breeding of Japanese Medaka (Oryzias Latipes)

**Figure 1:**
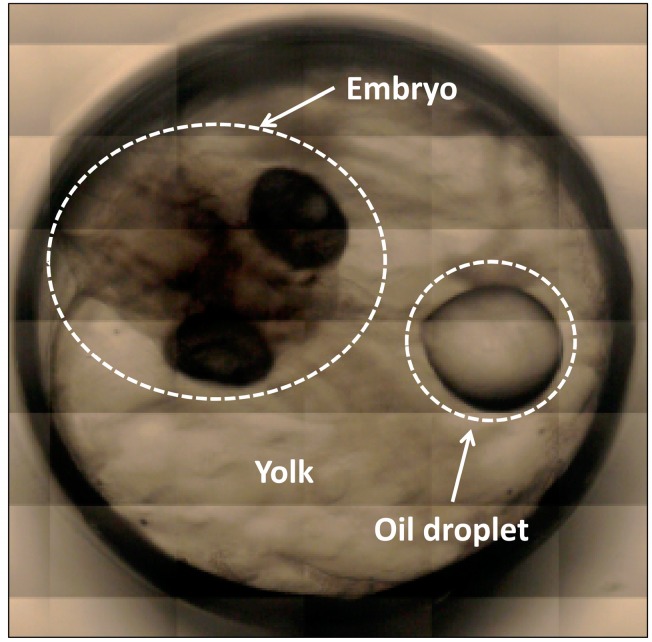
An optical image showing a fertilized medaka egg from the fifth day after fertilization taken with a microscope at 5× magnification.

Male and female medaka (*O. latipes*) fish were bred in fresh water at 25 °C, and were fed a commercial medaka fish feed. This fish species is often used as a laboratory model to test compounds for carcinogenicity [27,28]. The eggs produced were approximately 1.5 mm in diameter. They were transparent and suitable for the spectroscopic analysis. A standard egg hatches approximately two weeks after fertilization under normal feeding conditions [29,30]. In the unfertilized egg, a number of small oil droplets are visible, and soon after fertilization, the oil droplets that are distributed throughout the egg begin to coalesce and fuse into larger droplets. In regions where there are no large oil droplets present, cytoplasm accumulates to form a blastodisc and unequal cleavage takes place, which transforms into the embryonic body. The detailed development processes of this species are explained in ref. [29]. Figure 1 depicts an optical image showing a fertilized medaka egg on the fifth day after fertilization taken with a camera attached to a microscope at 5× magnification. On the day following fertilization, the embryonic body can easily be seen in the egg (Figure 1). The egg consists of three major parts: oil droplets, egg yolk, and embryo. The oil droplets are rich in lipids (unsaturated fatty acids), while the egg yolk contains both lipids and proteins [30,31]. The egg contains sufficient energy in the oil droplets and egg yolk to complete egg development until first feeding commences [32], with total lipid contents decreasing 30% during egg development [33]. The medaka eggs hatch approximately two weeks after fertilization, as mentioned previously; however, the period before hatching is variable. In the present study, the eggs hatched between 11 and 14 days after fertilization. Hence, it is necessary to define a method to be able to assign the data as being the previous day of hatching. In the majority of cases, the eggs spawned by one individual hatched on the same day. One egg was chosen from the eggs spawned by one individual and NIR measurement was performed. If the other eggs spawned by the same individual hatched on the next day after the original NIR measurement, the NIR data was assigned as JBH.

### 2.2. Instrumentation and NIR Measurements

The Fourier Transform (FT)-NIR spectral measurement and imaging measurement were performed using a Perkin Elmer (Waltham, MA, USA) imaging system consisting of a Spectrum One FT-NIR spectrometer coupled to a Spectrum Spotlight 300 NIR microscope. This totally integrated instrument incorporates a 16 element (400 × 25 μm^2^) HgCdTe (MCT) array detector and a single point 100 × 100 μm^2^ MCT detector in the same Dewar. The symmetrically arranged objective and condenser provide a 6× image magnification at 1:1 imaging, with a numerical aperture of 0.58. For the present study, each image was measured in the transmission mode over an area of 1.5 × 1.5 mm^2^ with a pixel size of 25 × 25 μm^2^ and a spectral resolution of 8 cm^−1^ by co-adding eight scans for a spectrum in 6200–4000 cm^−1^ region.

The eggs had an almost spherical form, therefore, to regulate the optical path length, NIR measurements were performed by sandwiching the egg between two glass slides using a pinchcock. Thus, the optical path length was set at 0.36 mm.

NIR spectra were measured in single point mode from three major parts: oil droplets, egg yolk, and embryo. The measurement time for one single point was approximately one minute. The vascular flow was confirmed within approximately 20 min measurements. Three eggs at each developmental stage from the first day after fertilization to the day JBH were selected. Ten points for each three parts of the egg were measured to reduce the individual variability and dependence of measurement points. After the measurements were taken, the averaged NIR spectra were calculated for each day and each part. The data on the second day were not referred to because the border between the blastodisc and egg yolk became temporarily unclear. The NIR imaging data, on the other hand, were obtained using imaging mode, taking approximately 40 min to obtain the imaging data. The images were drawn by plotting the second derivative intensities at the notable bands in two dimensions using Graph-R software.

To search for specific bands, the spectra were subjected to a second derivative procedure (11 points). Principal component analysis (PCA) was performed to extract the different factors between samples using the chemometrics software Unscrambler X 10.3 (Woodbridge, NJ, USA).

The present experiments were performed in accordance with the fundamental guidelines for proper conduct of animal experiments and related activities in academic research institutions under the jurisdiction of the Ministry of Education, Culture, Sports, Science, and Technology in Japan. The study was carried out with permission by the Ethics Committee of Kwansei Gakuin University.

## 3. Results and Discussion

### 3.1. NIR Spectroscopy Study

**Figure 2:**
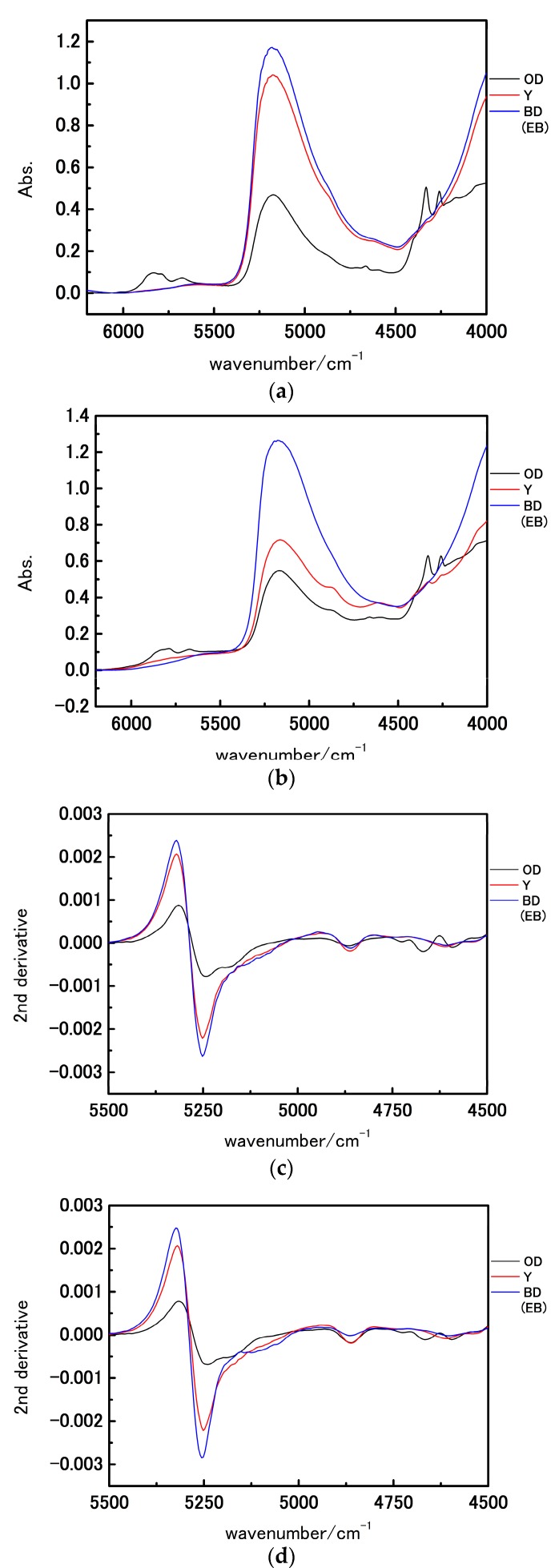

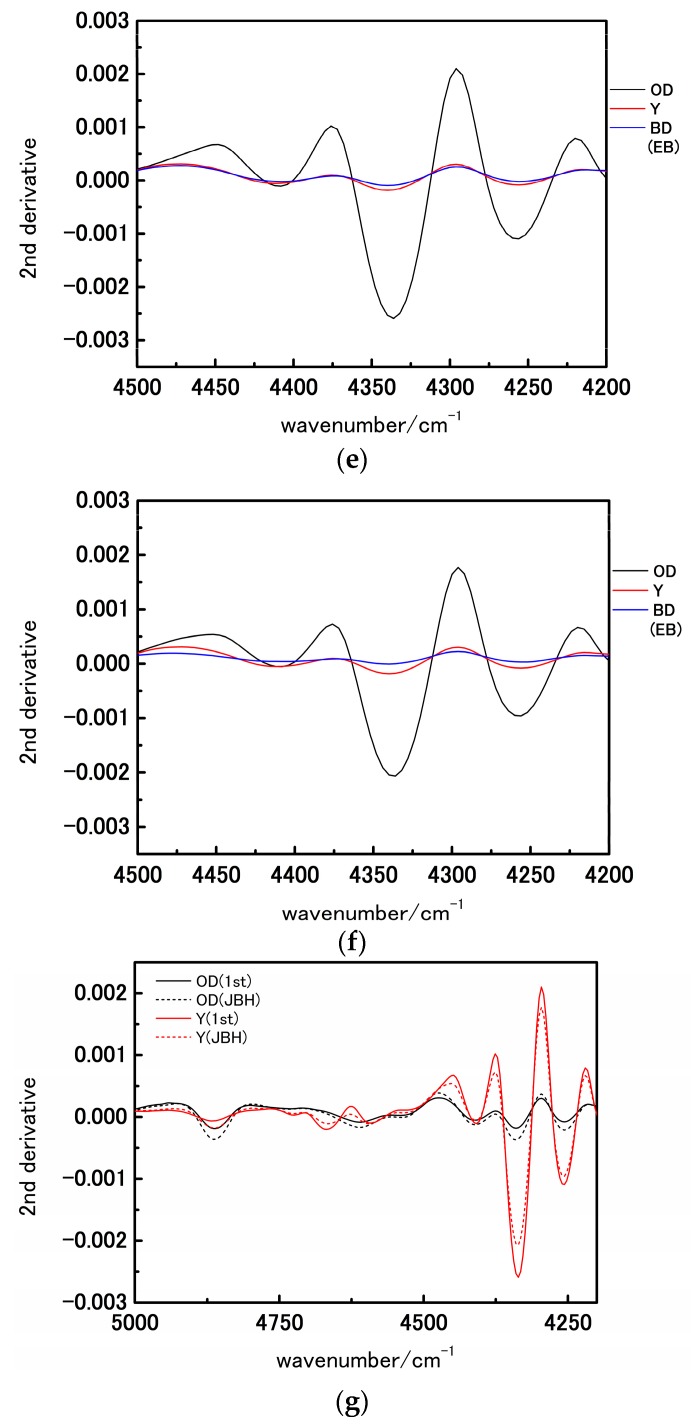
(**a**,**b**) NIR spectra in the 6200–4000 cm^−1^ region of the oil droplets, egg yolk, and blastodisc (embryonic body) on the fertilization day (first day) and day just before hatching (JBH); (**c**–**f**) are second derivatives of the spectra shown in Figure 2a,b in the 5500–4500 cm^−1^ and 4500–4200 cm^−1^ regions, respectively; (**g**) The overlay of the second derivative spectra in the 4900–4200 cm^−1^ region. (OD: oil droplets, Y: egg yolk, BD: blastodisc, EB: embryonic body).

Figure 2a,b show NIR spectra in the 6200–4000 cm^−1^ region of the three major parts (oil droplets, egg yolk, and embryo) in a fertilized egg of medaka on the first day after fertilization and the day JBH, respectively. All the spectra show a broad feature at approximately 5200 cm^−1^ owing to a combination of the antisymmetric OH stretching mode and OH bending mode of water. The spectra of oil droplets notably developed several strong bands in the 4350–4250 cm^−1^ region arising from lipids and the egg yolk in this region show weak corresponding bands. Figure 2c–f depict the second derivative spectra in the 5500–4500 cm^−1^ and 4500–4200 cm^−1^ region, respectively. The second derivative spectra yield weak features at 4864 and 4616 cm^−1^ owing to a combination of the NH stretching mode and amide II mode of egg proteins and two intense bands at approximately 4332 and 4260 cm^−1^ assignable to the combination of CH stretching and bending modes of lipids [34,35]. In Figure 2c,d the spectral shapes in the 5300–5200 cm^−1^ region are notably different between the spectra of oil droplets and the other parts. Sacis et al. [36] reported that an NIR spectrum of water gives two major bands at approximately 7700 and 6500 cm^−1^, which is assignable to water engaging in weaker and stronger hydrogen bonds, respectively. Regarding the water band at approximately 5250 cm^−1^, Czarnik-Matusewicz et al. [37] assigned two bands at approximately 5200 and 5000 cm^−1^ to weakly and strongly hydrogen-bonded water, respectively, using a three-component water model. The spectral shape in the 5300–5000 cm^−1^ region is different between oil droplets and other parts as shown in Figure 2c,d. The relative intensity of two bands at 5180 and 5260 cm^−1^ is higher in the oil droplets than in the other parts, which arises from the fact that the contribution rate from strongly and weakly hydrogen-bonded water is different depending on the parts of the egg.

Figure 2g shows the overlay of the second derivative spectra in 4900-4200 cm^−1^ region from oil droplets and egg yolk on the 1st day after fertilization and the day JBH. In oil droplets, the band intensities at 4332 and 4260 cm^−1^ owing to lipids were low and that from proteins at 4860 cm^−1^ band intensities were high (Figure 2g). Lipid components are utilized for embryonic development and lipid concentrations are assumed to decrease over time. A fish lives with only the nutrients stored in their abdomens until first feeding [32]. To be able to use proteins within the oil droplets as energy sources, these need to be decomposed into amino acids. The result that the second derivative intensity owing to NH stretching mode at 4864 cm^−1^ was high at the day JBH indicates that the concentration of amino acids increased to be utilized as an energy source after hatching. The present result may be useful in obtaining signs of metabolism changes.

With reference to the egg yolk elements, one noteworthy difference is seen in the water. The band intensity of water at approximately 5250 cm^−1^ diminished, and the egg yolk was seen to have a significantly different water environment from the other parts during development. Eventually, the second derivative spectra obtained from the embryonic body had higher intensity of water absorbance than did the other parts. These results indicate that the variations in the components concentrations with the development of the fish egg and its parts are reflected in the water structure. Furthermore, the water band at 5248 cm^−1^ on the first day after fertilization shifted to 5252 cm^−1^ on the day JBH, indicating that the fraction of weakly hydrogen-bonded water was increasing.

**Figure 3:**
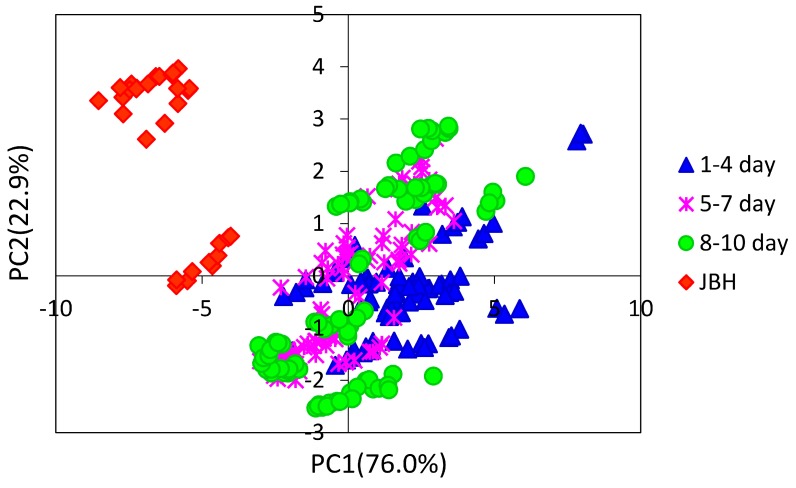
PC1 and PC2 score plot built using the NIR spectra of the egg yolk obtained in the period of the first day after fertilization to the day just before hatching (JBH).

**Figure 4:**
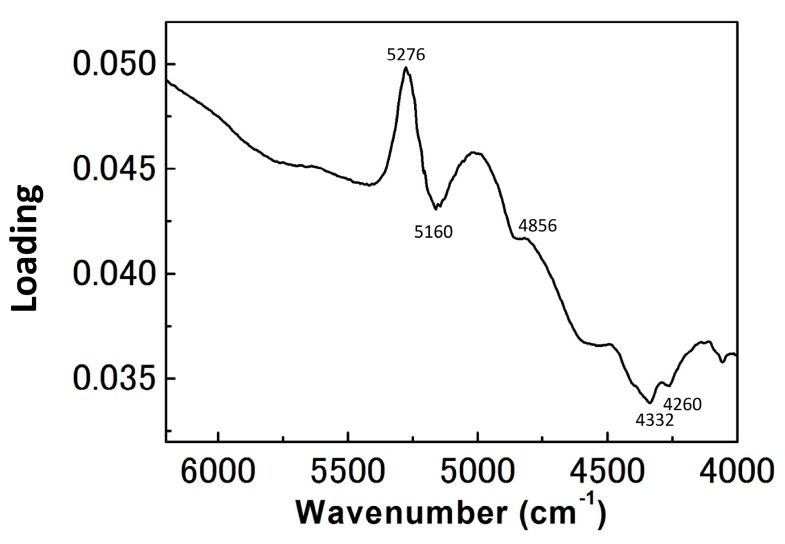
Loadings plot of PC1 of the PCA shown in Figure 3.

**Figure 5:**
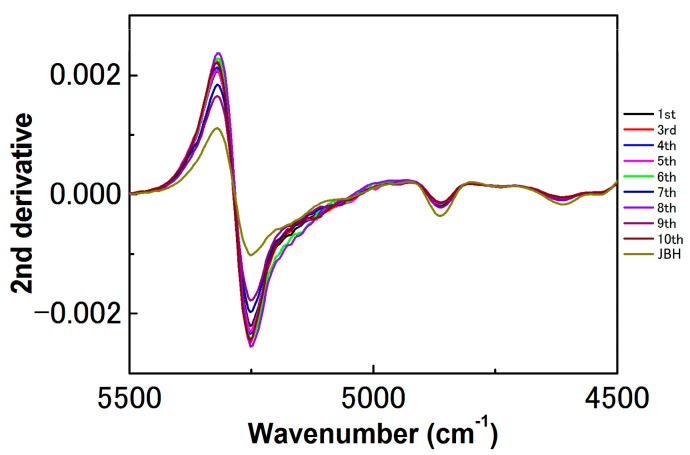
Second derivatives spectra in the region of 5500–4500 cm^−1^ of the egg yolk from the first day after fertilization to the day just before hatching (JBH).

**Figure 6:**
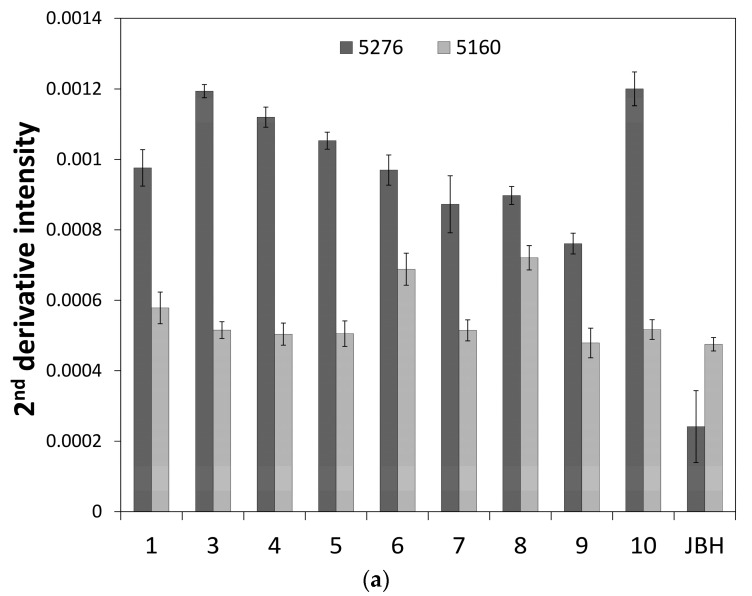

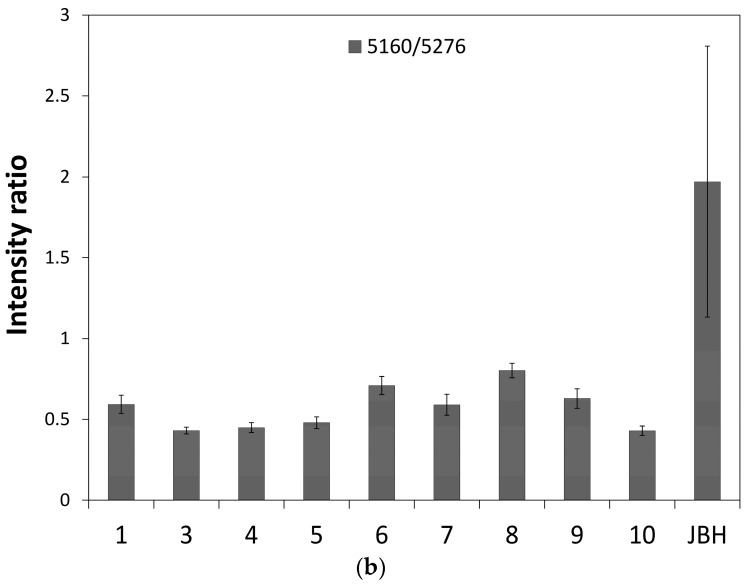
The plots of (**a**) the second derivative intensity with opposite signs at 5276 and 5160 cm^−1^ and (**b**) the intensity ratio of 5160/5276 cm^−1^ versus the number of days after fertilization.

Figure 3 depicts a PC1 and PC2 score plot of the PCA that was built using the NIR spectra of egg yolk obtained in the period from the first day after fertilization to the day JBH. Of note in Figure 3 is that the data obtained on the day JBH are clearly separated from other data in PC1. Figure 4 shows a loadings plot of PC1 of the PCA shown in Figure 3, with peaks at 5276 and 5160 cm^−1^ assigned to weakly hydrogen-bonded and strongly hydrogen-bonded water, respectively [36,37]. It is highly possible that the conditions of hydrogen bonding of water change significantly JBH. Figure 5 depicts variations in the 5500–4500 cm^−1^ region of the second derivative spectra of egg yolk measured in the period from the first day after fertilization to the day JBH. The intensity ratio of the two bands at 5276 and 5160 cm^−1^ caused by weakly hydrogen-bonded and strongly hydrogen-bonded water, respectively, were significantly different on the day JBH. The second derivative intensity with the sign inverse at 5276 and 5160 cm^−1^ can be seen in Figure 6a and the intensity ratio of 5160/5276 cm^−1^ versus day is shown in Figure 6b. The band intensity at 5160 cm^−1^ remained the same, but at 5276 cm^−1^ the band intensity diminished, with the intensity ratio increasing on the day JBH. Therefore, it is highly possible that the percentage of the weakly hydrogen-bonded water decreases markedly on the day JBH. In our previous study, we found that the ratio of the characteristic peaks owing to proteins and lipids in the second derivative spectra suggested that the relative concentration of proteins to lipids was constant in the egg yolk. However, the ratio of chemical components such as proteins and lipids to water increased in the present study, as shown in Figure 2c–f. Hence, water might interact strongly with the surrounding materials resulting in the weakly hydrogen-bonded water decreasing. The components variations and interaction changes between molecules toward hatching are assumed to be reflected in the water hydrogen bonding structure.

**Figure 7:**
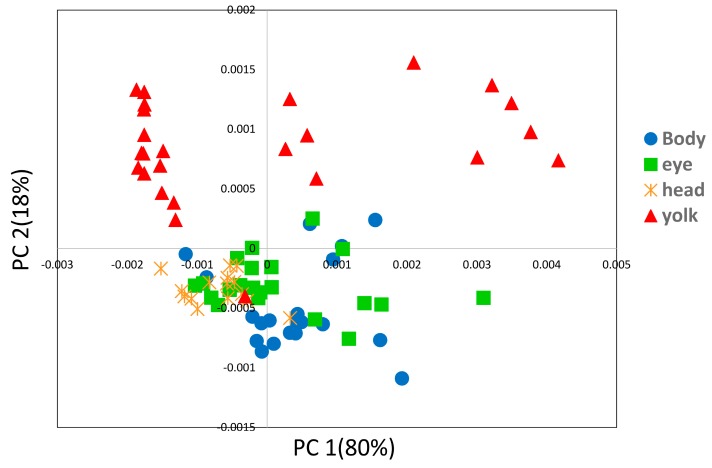
PC1 and PC2 plot of the PCA built by using second derivative spectra of the egg yolk and embryo obtained in the period of the first day after fertilization to the day just before hatching (JBH).

We performed PCA using NIR second derivative data of the egg yolk and embryo obtained from the egg on the fifth day after fertilization. The spectra from the embryo occasionally structurally overlapped with the spectra from the egg yolk and embryo. By analyzing the differences between the egg yolk and embryo data, it is possible to extract clearly the characteristic components for each part. Figure 7 shows the PC1 and PC2 plot of the PCA, and it can be seen that the egg yolk and other parts of the embryo are clearly separated into two groups.

**Figure 8:**
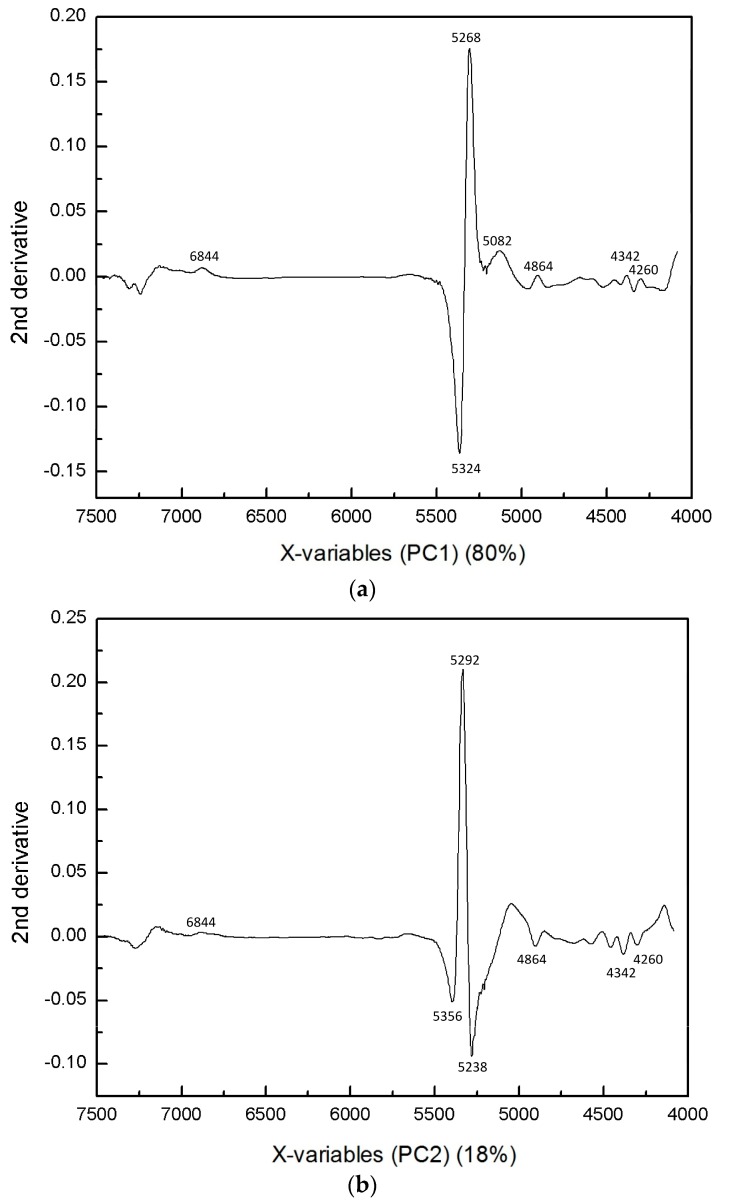
Loadings plots of (**a**) PC1 and (**b**) PC2 of the PCA shown in Figure 7.

Figure 8a,b depict PC1 and PC2 loading plots of the PCA shown in Figure 7. In the PC2 loading plot, sharp peaks are observed in the 5350–5250 cm^−1^ region. These peaks are assigned to water, suggesting that the egg yolk has a relatively different water environment to the embryo. The fact that the intensity at 5292 cm^−1^ in the second derivative spectra had a large value for the egg yolk implies that the egg yolk had less weakly hydrogen-bonded water than the embryo body, which may reflect the differences in the components concentrations, i.e., the inner components concentrations in the egg yolk are a high energy source. Furthermore, PC2 also had several bands owing to proteins and hydrocarbons, which indicates the chemical variations with the growth of fertilized eggs and the different composition distributions of materials in each section of the embryo.

### 3.2. Imaging Study

**Figure 9:**
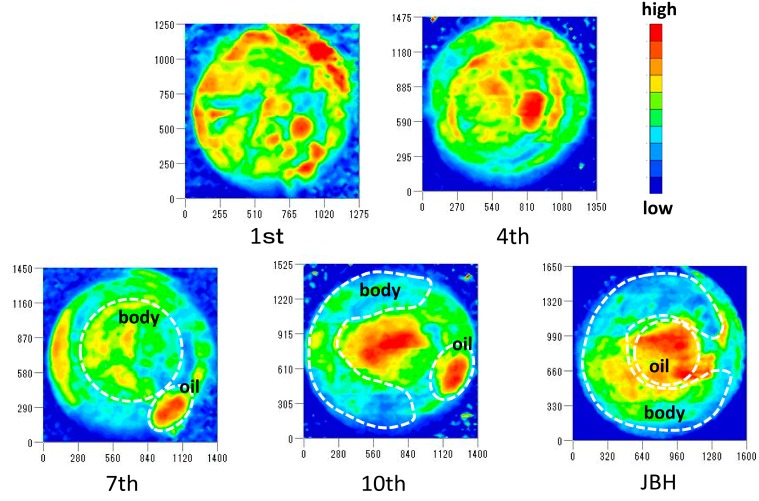
NIR images developed by using the intensity ratio of 5160/5276 cm^−1^ in absorbance spectra with the egg development.

Figure 9 displays the NIR images developed by using the intensity ratio of 5160/5276 cm^−1^ in absorbance spectra and shows the different distributions of weakly hydrogen-bonded water. These images show that the embryonic parts and oil droplet parts have high and low intensity ratios, respectively, which means that the embryo parts include a greater amount of weakly hydrogen-bonded water, while the oil droplet parts have strongly hydrogen-bonded water. The imaging results are consistent with the other discussions above.

**Figure 10:**
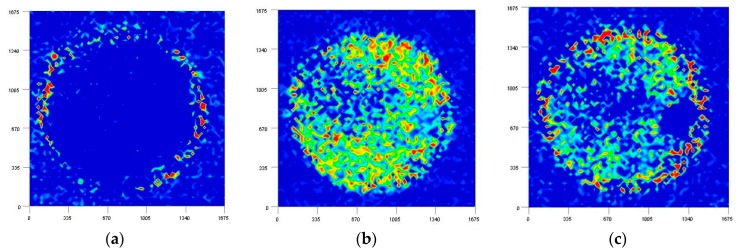

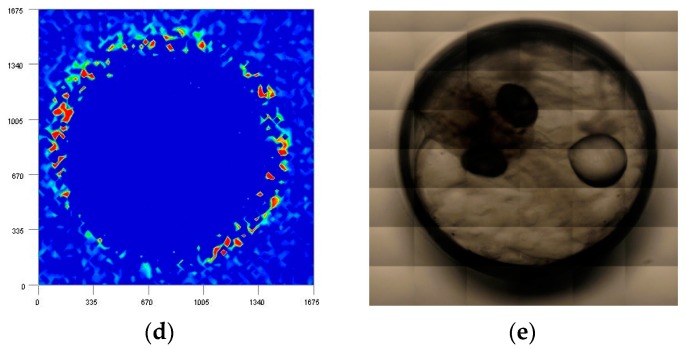
NIR imaging of fertilized medaka eggs obtained by using the intensity at (**a**) 5756; (**b**) 4864; (**c**) 4616; (**d**) 4530 cm^−1^; and (**e**) visible image.

Figure 10 compares the NIR images developed by the intensities of the second derivative spectra at (a) 5756; (b) 4864; (c) 4616; and (d) 4530 cm^−1^ caused by proteins in a medaka egg on the fifth day after fertilization. According to the literature, these bands are assigned to (a) α-helix; (b) combination of the NH stretching mode; (c) amide II; and (d) β-sheet structure, although there is still room for further research to assign the bands for the membrane structure and protein secondary structures [38,39]. From Figure 10b,c it can be seen that the egg yolk contains higher concentrations of proteins than do the other parts. It is assumed that 5756 and 4530 cm^−1^ are characteristic peaks from proteins that consist of membranes as shown in Figure 10a,d. Previous studies have indicated that proteins forming membranes have an almost α-helix and β-barrel secondary structure [40,41]. The fact that the membrane structure of the egg is successfully visualized by NIR imaging, as shown in the present study, is an important result.

## 4. Conclusions

In the present study, the variation, localization, and concentration gradient of the medaka fish egg’s interior components were successfully detected using NIR spectroscopy and NIR imaging. The present study has demonstrated one application of NIR spectroscopy and imaging for the in situ monitoring tool of egg growth for both basic studies and practical applications. We were able to monitor relative variations of weakly hydrogen-bonded and strongly hydrogen-bonded water among the egg’s interior components. The variations in components species and concentration depending on the spatial distribution or with egg development caused the differences in the interactions between the molecules of the components. These effects generate water structure variations. In conclusion, life events occurring within the egg during development were shown to have an impact on the hydrogen bonding of water. New imaging data developed from intensities at 4864 and 4616 cm^−1^ in the second derivative spectra arising from a combination of the NH stretching mode and amide II have demonstrated clearly different distributions of the proteins. Of particular note was that the images drawn with the bands from characteristic secondary structure of proteins at 5756 and 4530 cm^−1^ exhibited membrane structures.
